# Stimuli of Sensory-Motor Nerves Terminate Arterial Contractile Effects of Endothelin-1 by CGRP and Dissociation of ET-1/ET_A_-Receptor Complexes

**DOI:** 10.1371/journal.pone.0010917

**Published:** 2010-06-01

**Authors:** Merlijn J. P. M. T. Meens, Matthijs G. Compeer, Tilman M. Hackeng, Marc A. van Zandvoort, Ben J. A. Janssen, Jo G. R. De Mey

**Affiliations:** 1 Department of Pharmacology and Toxicology, Cardiovascular Research Institute Maastricht (CARIM), Maastricht University, Maastricht, The Netherlands; 2 Department of Biochemistry, Cardiovascular Research Institute Maastricht (CARIM), Maastricht University, Maastricht, The Netherlands; 3 Department of Biomedical Technology, Cardiovascular Research Institute Maastricht (CARIM), Maastricht University, Maastricht, The Netherlands; Hong Kong University, Hong Kong

## Abstract

**Background:**

Endothelin-1 (ET-1), a long-acting paracrine mediator, is implicated in cardiovascular diseases but clinical trials with ET-receptor antagonists were not successful in some areas. We tested whether the quasi-irreversible receptor-binding of ET-1 (i) limits reversing effects of the antagonists and (ii) can be selectively dissociated by an endogenous counterbalancing mechanism.

**Methodology/Principal findings:**

In isolated rat mesenteric resistance arteries, ET_A_-antagonists, endothelium-derived relaxing factors and synthetic vasodilators transiently reduced contractile effects of ET-1 but did not prevent persistent effects of the peptide. Stimuli of peri-vascular vasodilator sensory-motor nerves such as capsaicin not only reduced but also terminated long-lasting effects of ET-1. This was prevented by CGRP-receptor antagonists and was mimicked by exogenous calcitonin gene-related peptide (CGRP). Using 2-photon laser scanning microscopy in vital intact arteries, capsaicin and CGRP, but not ET_A_-antagonism, were observed to promote dissociation of pre-existing ET-1/ET_A_-receptor complexes.

**Conclusions:**

Irreversible binding and activation of ET_A_-receptors by ET-1 (i) occur at an antagonist-insensitive site of the receptor and (ii) are selectively terminated by endogenously released CGRP. Hence, natural stimuli of sensory-motor nerves that stimulate release of endogenous CGRP can be considered for therapy of diseases involving ET-1.

## Introduction

Prototypic G-protein coupled receptors (GPCR) are characterized by tight agonist concentration-response relationships on the short run and by tolerance on the long run. For instance, acute β_2_-adrenoceptor stimulated cAMP production and the resulting smooth muscle relaxation are readily reversible as a result of rapid dissociation of the agonist-receptor complexes. This property underlies the therapeutic applicability of drugs that inhibit the synthesis or the receptor-binding of endogenous GPCR-agonists. During prolonged exposure to agonists, β_2_-adrenergic effects fade as a result of phosphorylation, desensitization, uncoupling from the G-proteins and internalization of the receptors (for review see [1]).

In sharp contrast, the GPCR-agonist endothelin-1 (ET-1) causes long-lasting effects. Its in vitro arterial contractile effects persist after thorough washout of the agonist[2]. Its in vivo vasopressor effects are maintained long after clearance of the peptide from the circulation by the lungs and the kidneys[3]. The 21 amino acid bicyclic peptide, that is constitutively expressed by the endothelium and that can be induced in several other cell types[4], [5], is implicated in several cardiovascular diseases[4], [6], [7], cancers[8] and pain[9]. Its vasoconstrictor, pro-inflammatory, oxidative and mitogenic effects are mediated by ET_A_-receptors[4], [6], [7] while more beneficial effects such as endothelium-dependent vasodilatation and scavenging of circulating ET-1 are mediated by distantly related ET_B_-receptors[4], [5], [6], [7], [10]. ET_B_-agonism can be mimicked by short C-terminal fragments of ET-1[11], [12], [13] but high affinity ET_A_-agonism requires the full length, both disulfide bonds and distinct amino acids in the N-terminal loop of the peptide[12], [14], [15], [16], [17], [18]. This suggests that distinct parts of ET-1 have different functions in binding and activation of ET_A_-receptors. Several classes of low molecular weight ET_A_-selective or mixed ET-receptor antagonists have been developed primarily on the basis of prevention of the binding of ET-1 to its receptors[4], [5], [6], [19], [20], [21]. These compounds are thought to compete with the C-terminal tail of the agonist. They can prevent ET-1-induced effects in vitro (for review see [2]) and in animal studies[4], [6], [19]. They are, however, less effective in reversing the effects of ET-1 in vitro[2], in animal studies[22] and in clinical trials[6], [23]. This may be due to the atypical properties of ET_A_-receptors.

Irreversible agonism by ET-1 is incompatible with homeostasis unless counterbalancing systems exist. ET-1 can stimulate NO release from the endothelium[24]. NO reduces ET-1 synthesis[25] and counteracts vasoconstriction initiated by ET_A_-receptors on smooth muscle cells[4], [7], [26]. ET-1 can also promote activity of transient receptor potential (TRP) cation channels that stimulate release of vasodilator neurotransmitters from peri-arterial sensory-motor nerves (SMN)[27], [28]. Hence, in cardiovascular diseases characterized by reduced bioavailability of endothelium-derived NO, ET-1 and ET_A_-effects are upregulated[4] and can be tempered by counterbalancing effects of SMN[29], [30], [31]. Whether the latter involves functional antagonism or a selective effect on ET_A_-receptors has not been addressed.

In this study, we hypothesized that polyvalent agonist-receptor binding by ET-1 limits reversing effects of ET-receptor antagonists and used physiological reasoning to search for a superior inhibitor. For these purposes we studied rat mesenteric arteries in which ET_A_- and ET_B_-receptors are expressed by several cell types[31], [32], [33]. We discovered that calcitonin-gene related peptide (CGRP) released from peri-arterial SMN terminates long-lasting vasoconstrictor effects of ET by promoting dissociation of ET-1/ET_A_-receptor complexes.

## Results

### Key role of smooth muscle ET_A_-receptors in long-lasting arterial contractile responses to ET-1

**Figure 1:**
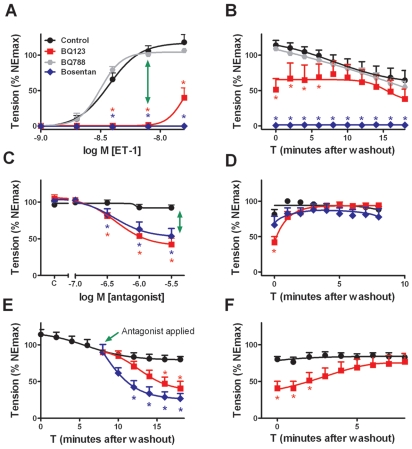
Partial and reversible reversing effect of ET-receptor antagonists on arterial contractile responses to ET-1 and their persistence. Isolated rat mesenteric resistance arteries were studied after treatment with capsaicin and in continuous presence of L-NAME (100 µM) and indomethacin (10 µM). A, responses to 0.25–16 nM ET-1 in absence (black) and presence of BQ123 (1 µM, red), BQ788 (1 µM, grey) or bosentan (3 µM in presence of 1 µM BQ788, blue). BQ123 and bosentan prevented responses to up to 8 nM ET-1. B, vasomotor tone after removal of free agonist and antagonist. C, effects of BQ123 and bosentan (0.1–3.0 µM) in the presence of 8 nM ET-1. D, vasomotor tone after removal of free agonist and antagonist. E, effect of BQ123 (1 µM) and bosentan (3 µM) on contractile responses initiated by 8 nM ET-1 that persisted in absence of the peptide. F, vasomotor tone after removal of free antagonist. n = 6–20. *, P<0.05 vs. control.

In isolated rat mesenteric resistance arteries, the ET_B_-selective agonist Ala^1,3,11,15^-ET-1[10] (1 nM–1 µM) caused neither contraction (Table 1) nor relaxation (data not shown). In contrast, the non-selective agonist ET-1[10] potently stimulated contractions (Fig. 1A, table 1). The concentration-response relationship was steep and the responses were quasi-irreversible (T_1/2_ >20 min versus T_1/2_ ≈30 sec for similarly strong contractile responses to norepinephrine (NE) (Fig. 1A/B). Contractile effects of ET-1 and their persistence were not modified by 1 µM BQ788 (ET_B_-antagonist)[10], [34], 100 µM L-NAME and 10 µM indomethacin (which reduce endothelial influences), nor by pre-treatment with capsaicin (1 µM during 20 min, which reduces effects of SMN) (Fig. 1A and table 1). Mechanical removal of the endothelium resulted in a small increase in the sensitivity for ET-1 (Table 1). The sensitivity to ET-1 was reduced in presence of the ET_A_-antagonists BQ123[10], [35] (1 µM), SB234551[10], [21] (10 nM) or bosentan[10], [19] (3 µM studied in presence of 1 µM BQ788 to focus on ET_A_-antagonism by boseantan, a mixed ET_A/B_-receptor antagonist) (Fig. 1A, Fig. S1A).

**Table 1:** Arterial effects of ET-1 and two analogues.

Agonist	Condition	EC_50_ (nM)	E_max_ (% NE_max_)	Tension (%NE_max_; 8 min after agonist removal)
ET-1	-	4.9±0.8	88.5±4.0	80.9±3.5
	Denuded	2.1±0.2 *	100.1±6.2	82.54±5.4
	L-NAME + INDO	3.5±0.5	100.4±5.0	79.2±3.3
	CAPS + L-NAME + INDO	3.6±0.3	104.0±4.0	84.5±14.0
Rh-ET-1	CAPS + L-NAME + INDO	4.1±0.3	102.0±10.0	80.6±14.6
Ala^1,3,11,15^-ET-1	CAPS + L-NAME + INDO	>1 µM	0	0

Potency, efficacy and persistence (response remaining at 8 min after agonist removal) are shown for arteries without and with pre-treatment with capsaicin (1 µM, 20 min; CAPS) and presence of L-NAME (100 µM) and indomethacin (10 µM; INDO) and for denuded arteries. n = 6–10. *: p<0.05 vs control.

### Partial and transient reversing effects of ET_A_-antagonists

Although BQ123, SB234551 and bosentan prevented contractile responses to up to 8 nM ET-1, the antagonists could only partly (≈50%) reverse contractile responses initiated by 8 nM ET-1 (Fig. 1C, Fig. S1C). The relaxing effect of the antagonists was reversible, i.e. vasomotor tone rapidly recovered after washout of the ET-receptor ligands (Fig. 1D, Fig. S1D). This indicates irreversible agonism and reversible antagonism. In addition, contractile effects of ET-1 that persisted in absence of free agonist were partly and transiently reduced by the antagonists (Fig. 1E/F, Fig. S1E/F).

### Transient reversing effects of endothelium-derived and exogenous vasodilators

**Figure 2:**
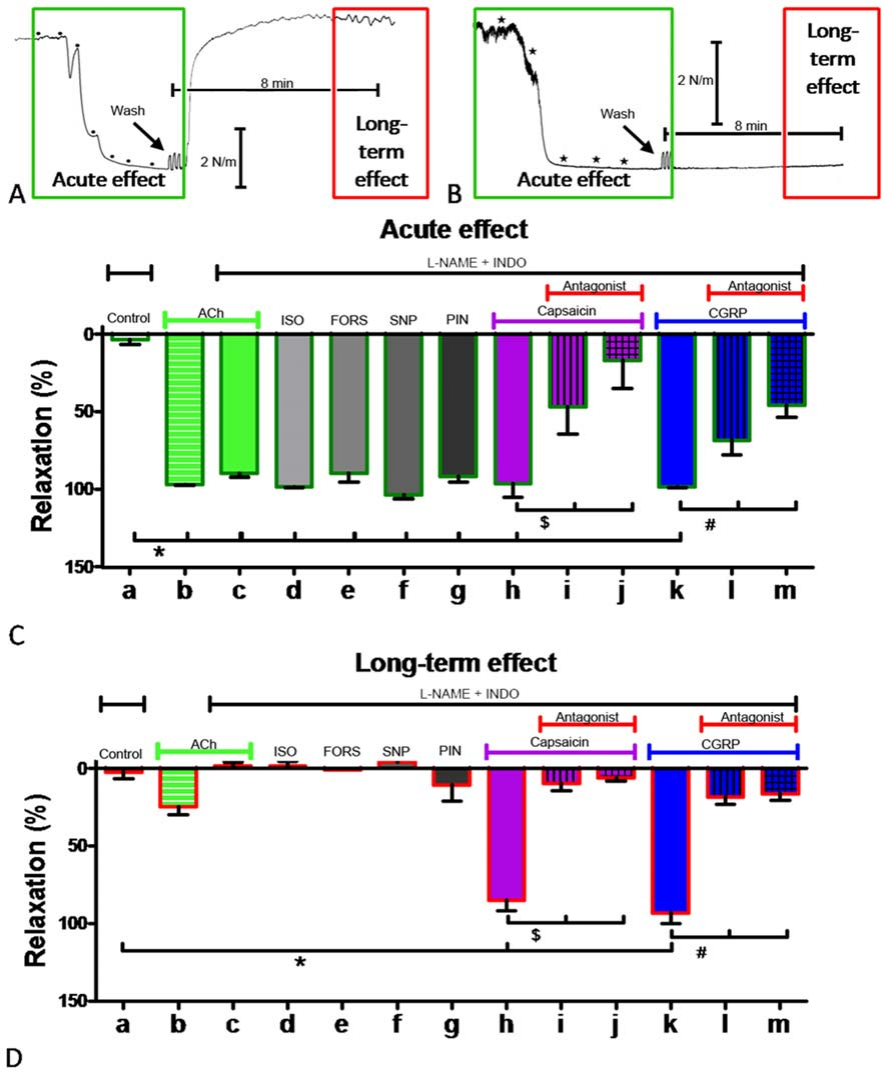
Capsaicin and CGRP relax endothelinergic arterial contraction and prevent the persistent contractile effect of ET-1. Isolated rat mesenteric resistance arteries were studied in presence of L-NAME (100 µM) and indomethacin (10 µM) (as indicated) and were contracted with ET-1 (16 nM). Increasing vasodilator concentrations were administered until a maximal effect was observed. Thereafter vasoconstrictor and vasodilator stimuli were removed from the organ chamber while the recording of active wall tension continued for >10 min. A and B, typical tracings of active wall tension (WT) versus time (t) illustrating acute relaxing effects (green box) of acetylcholine (A; 0.01–10 µM) and capsaicin (B; 0.01–1 µM) and rapid recovery of contraction after removal of the vasodilator (long-term effect, red box) in the case of acetylcholine (A) but not capsaicin (B). C, maximal acute relaxing effects of various dilators. D, long-term effects of various dilators. a, time control; b and c, acetylcholine; d, isoproterenol; e, forskolin; f, Na-nitroprusside; g, pinacidil; h – j, capsaicin in the absence (h) and presence of CGRP_8-37_ (i) or BIBN4096BS (j); k - m, CGRP in the absence (k) and presence of CGRP_8-37_ (l) or BIBN4096BS (m). For concentrations of vasodilators see “Methods” section. n = 6–8. *, $ and #: P<0.05 vs. control, capsaicin or CGRP, respectively.

**Figure 3:**
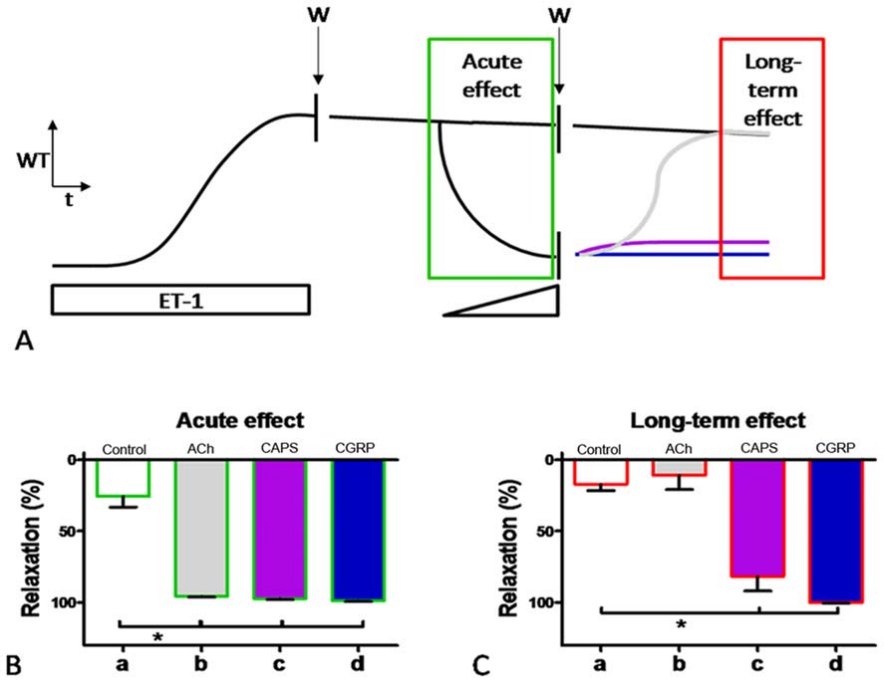
Capsaicin, CGRP and acetylcholine relax endothelinergic arterial contraction that remained after removal of ET-1 from its biophase but only capsaicin and CGRP prevent the persistent contractile effect of ET-1. A, schematic tracings of active wall tension (WT) versus time (t) illustrating i) ET-1-induced contractions that are not reversed upon agonist removal (W), ii) acute effects of various dilators and iii) prevention of long-term ET-1 effects by capsaicin (purple) and CGRP (blue) but not acetylcholine (grey). B, maximal acute relaxing effects of acetylcholine (b), capsaicin (c) or CGRP (d). C, long-term effects of acetylcholine (b), capsaicin (c) or CGRP (d). For concentrations of vasodilators see “Methods” section. n = 6–8. *: P<0.05 vs. control.

In contrast to ET-antagonists, several vasodilator stimuli fully reversed contractile responses to ET-1 (Fig. 2A). This was the case for acetylcholine (endothelium-dependent vasodilator), forskolin (direct activator of adenylyl cyclase), isoproterenol (beta-adrenergic stimulus of adenylyl cyclase), Na-nitroprusside (NO-donor) and pinacidil (activator of K_ATP_-channels) (Fig. 2C). However, vasomotor tone rapidly recovered in absence of vasodilators and ET-1 (Fig. 2D). Moreover, contractions remaining after exposure to ET-1 could be relaxed by for instance acetylcholine (Fig. 3B) but again this inhibitory effect was reversible (Fig. 3C).

### Effects of TRP-channel activators and CGRP

**Figure 4:**
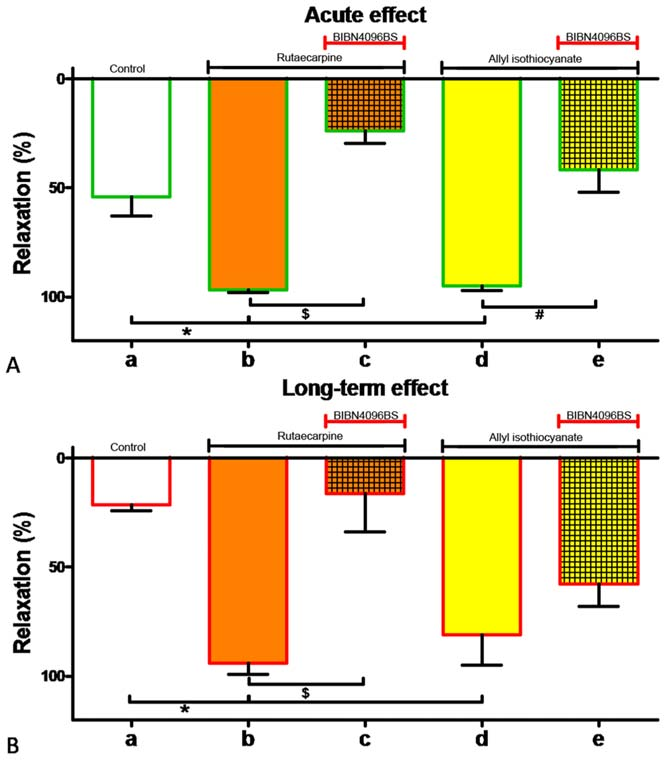
Stimuli of SMN, like rutaecarpine and allyl isothiocyanate, relax endothelinergic arterial contraction and prevent the persistent contractile effect of ET-1. Isolated rat mesenteric resistance arteries were precontracted with 16 nM ET-1. Increasing concentrations of vasodilator compounds were administered until a maximal effect was observed. Thereafter vasoactive stimuli were removed from the organ chamber while the recording of active wall tension continued for >10 min. A, maximal acute relaxing effects of rutaecarpine and allyl isothiocyanate. B, long-term effects of rutaecarpine and allyl isothiocyanate. a, time control; b, rutaecarpine; c, rutaecarpine in presence of BIBN4096BS; d, allyl isothiocyanate; e, allyl isothiocyanate in presence of BIBN4096BS. For concentrations of vasodilators see “Methods” section. n = 6–8. *, $ and #: P<0.05 vs. control, rutaecarpine and allyl isothiocyanate, respectively.

In contrast to these vasodilators, capsaicin relaxed ET-1-induced contractions (Fig. 2B/C) and prevented their recovery (Fig. 2B/D). This was also observed with rutaecarpine and with allyl isothiocyanate (Fig. 4A/B). In the case of rutaecarpine these effects were endothelium independent (Fig. S2). Capsaicin, rutaecarpine and allyl isothiocyanate stimulate release of several neurotransmitters from SMN[27], [36], [37], [38], [39], [40]. The CGRP-receptor antagonists CGRP_8-37_ [41] (1 µM) and BIBN4096BS[42] (20 nM)) reduced both the relaxation and the prevention of persistent effects of ET-1 by the SMN stimuli (Fig. 2C/D, Fig. 4A/B). Moreover, exogenous CGRP relaxed ET-1-induced contractions (Fig. 2C), prevented recovery of contractions initiated by ET-1 (Fig. 2D) and caused long-lasting inhibition of the persistent effects initiated by ET-1 (Fig. 3A/B). These effects were endothelium independent (Fig. S2) and were reduced by CGRP-receptor antagonists (Fig. 2C/D). The contraction that persisted after exposure to ET-1 and that was transiently inhibited by ET-receptor antagonists (Fig. 1E/F) or by acetylcholine (Fig. 3B/C), was terminated by capsaicin and by CGRP (Fig. 3B/C).

**Figure 5:**
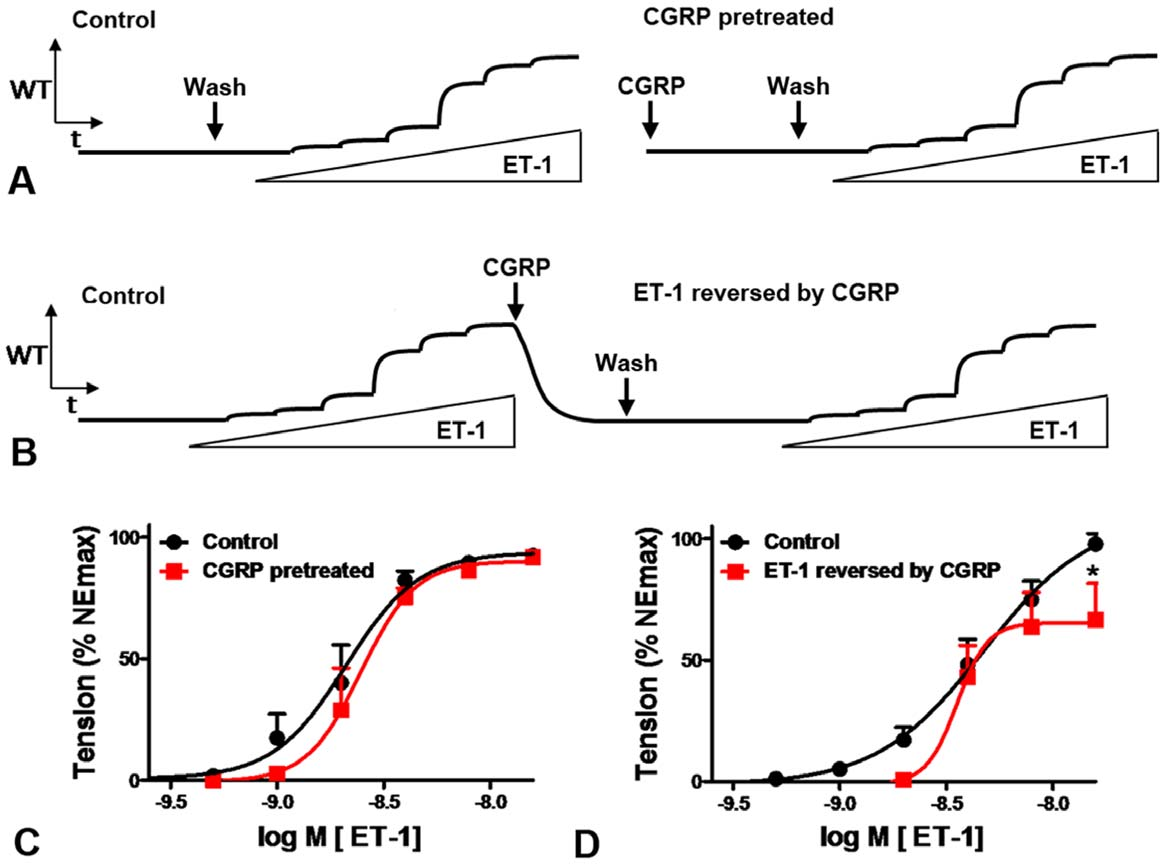
Unaltered arterial contractile responses to ET-1 (0.25–16 nM) following exposure to CGRP. A, schematic tracings of active wall tension (WT) versus time (t) illustrating contractile reponses to ET-1 (0.25–16 nM) in arteries transiently treated with (right) or without (left) CGRP (100 nM). B, schematic tracing of active wall tension (WT) versus time (t) illustrating initial ET-1 effects which were reversed by CGRP before a second concentration response curve was generated. C: Effect of ET-1 (0.25–16 nM) in arteries pre-treated with CGRP (100 nM, during 16 min). D: Effect of ET-1 (0.25–16 nM) in arteries in which ET-1-induced contractions were reversed by CGRP (100 nM). n = 6. *: p<0.05 vs control.

When arteries were transiently exposed to a high concentration of CGRP (100 nM; Fig. 5A) or to ET-1 (16 nM) and then to CGRP (100 nM; Fig. 5B), exogenous ET-1 (1–16 nM; applied after removing other vasoactive compound from the organ bath) caused contractions with a potency and an efficacy that deviate only marginally from those observed in controls (Fig. 5C/D). This suggests that CGRP does not induce a long-lasting relaxing effect (Fig. 5C) but rather promotes dissociation of previously established ET-1/ET_A_-receptor complexes allowing re-application of ET-1 to again induced contractile responses (Fig. 5D).

### Modulation of ET-1/ET_A_-receptor binding

**Figure 6:**
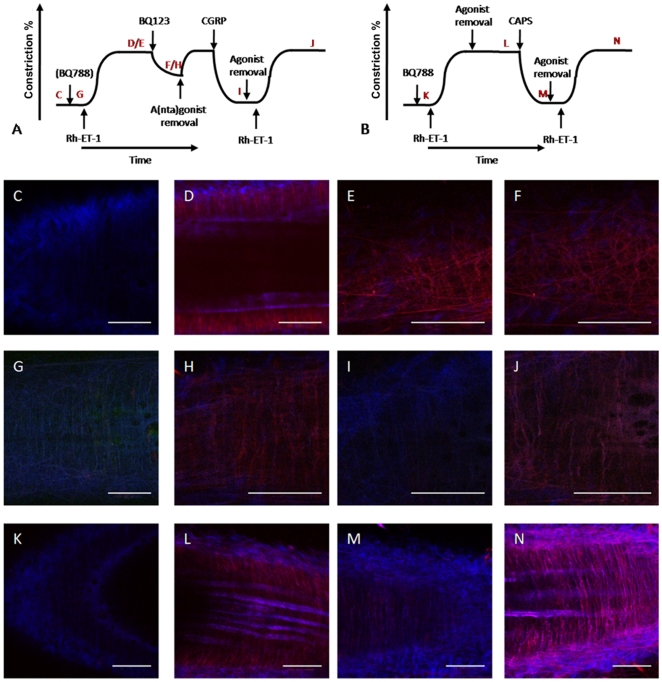
Binding of ET-1 is not reversed by ET-receptor antagonists (C-F) but can be reversed by CGRP (G-J) and by capsaicin (K-N). Isolated rat mesenteric arteries were canullated, pressurized and mounted under a 2-photon laser scanning microscope. Analyses focussed on the smooth muscle layer (bordered by the autofluorescent (blue) internal and elastic laminae) (C, G, H)). Experiments were performed in continuous presence of BQ788 (1 µM) except panels C and D. A and B illustrate schematic tracings of active wall tension versus time illustrating the order of (i) administration of rhodamine-labeled ET-1 (Rh-ET-1, 16 nM), (ii) application of pharmacological agents and (iii) removal of agonists and antagonists. C, autofluoresence. D - F, labeling of vascular smooth muscle (D, red) observed in presence of Rh-ET-1 is not noticeably affected by administration of BQ788 (E, 1 µM) and BQ123 (F, 1 µM). G, autofluorescence. H – J, labeling induced by exposure to Rh-ET-1 (16 nM) persists in absence of free label and is resistant to ET-receptor antagonists (H) but is rapidly abolished (I) by exposure of the artery to CGRP (100 nM); thereafter labeling of smooth muscle can be re-established by exposure to Rh-ET-1 (16 nM) (J). K, autofluoresence. L – N, largely similar experiment using capsaicin (CAPS, 1 µM). Labeling induced by exposure to Rh-ET-1 (16 nM) that persists in absence of free label (L) is abolished (M) by exposure of the artery to CAPS (1 µM); thereafter labeling of smooth muscle can be re-established by exposure to Rh-ET-1 (16 nM) (N). Scale bars: 50 µm. Findings are representative for 4 arteries of 3 rats.

We used rhodamine-labeled ET-1 (Rh-ET-1) and two-photon laser scanning microscopy (TPLSM) focusing on the tunica media, to visualize binding of ET-1 to the smooth muscle. Contractile properties did not differ between Rh-ET-1 and ET-1 (Table 1). Binding of Rh-ET-1 (16 nM) to smooth muscle (Fig. 6D) was reduced by BQ788 (1 µM; Fig. 6E) and was prevented by presence of either ET-1 (16 nM) or of both BQ788 (1 µM) and BQ123 (1 µM)[2] indicating selective binding to ET_A_- and ET_B_-receptors. Once established, binding of Rh-ET-1 persisted after washout of free Rh-ET-1 and was not reversed by BQ123 (1 µM; Fig. 6F) indicating quasi-irreversible receptor-binding of the agonist. In contrast, capsaicin (1 µM) and exogenous CGRP (100 nM; investigated in presence and absence (not shown) of 1 µM BQ788), reversed the binding of Rh-ET-1 to smooth muscle that remained after exposure to Rh-ET-1 (Fig. 6I/M). Thereafter, Rh-ET-1 could again label the arterial smooth muscle (Fig. 6J/N).

## Discussion

The novel finding of our work is that while ET-receptor antagonists partly and transiently reduce endothelinergic vasoconstriction as a result of bitopic and irreversible agonist-receptor binding, stimuli of SMN can terminate effects initiated by ET-1 through CGRP-receptors that promote dissociation of ET-1/ET_A_-receptor complexes. This may lead to novel therapies of diseases involving ET-1.

We compared effects and mechanisms of action of competitive and physiological antagonists of ET-1 in isolated rat mesenteric resistance arteries. In these vessels, which influence local blood flow and total peripheral resistance and contribute to the development of hypertension[43], ET-receptor subtypes are expressed by several cell types[31], [32], [33]. However, a selective ET_B_-agonist did not modify vasomotor tone. Contractile effects of ET-1 were not modified by an ET_B_-antagonist, pre-treatment with capsaicin or inhibition of NO-synthases and cyclo-oxygenases. Thus, initiation and maintenance of contractile responses to ET-1 were dominated by smooth muscle ET_A_-receptors and were hardly affected by basal or endothelinergic influences of SMN or the endothelium.

**Figure 7:**
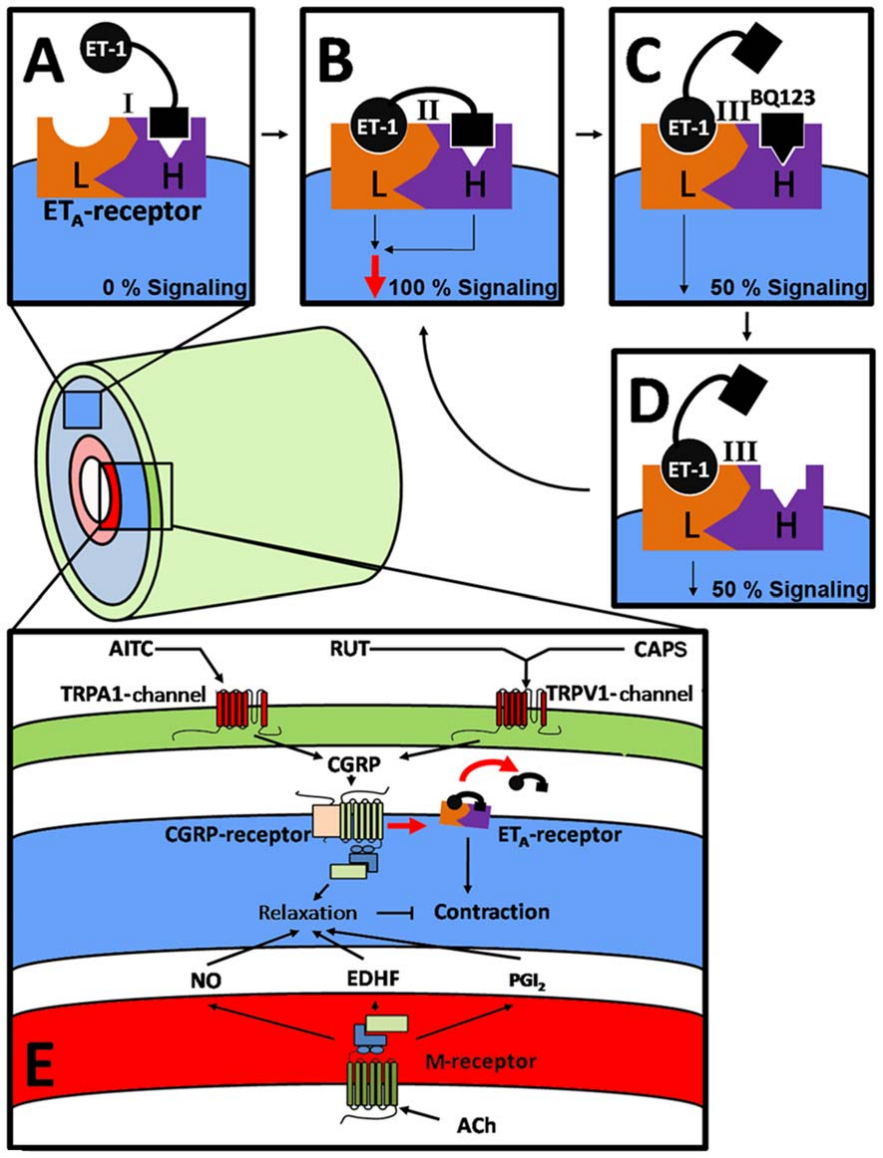
Schemes illustrating the interaction of ET-1 with arterial smooth muscle ET_A_-receptors (A – D) and the influences of vasodilators. A, Initially, a part of ET-1 binds to a high affinity binding site (H) on an ET_A_-receptor. B, next, another part of ET-1 binds to a low affinity binding site (L) of the receptor. Signaling is triggered by the occupied site L and reinforced by the occupied site H. C, while binding of ET-1 to site L is quasi-irreversible, the binding of ET-1 to site H remains dynamic and can be competed off by low molecular weight antagonists such as BQ123. D, binding of antagonists is readily reversible after which bivalent binding of ET-1 to the ET_A_-receptor and cooperative signaling can be re-established. E, schematic representation of the endothelium (red), arterial smooth muscle (blue) and peri-arterial SMN (green). Endothelium-derived relaxing factors such as nitric oxide (NO), endothelium-derived hyperpolarizing factor (EDHF) and prostacyclin (PGI_2_) released upon stimulation of e.g. endothelial muscarinic receptors (M) by acetylcholine (ACh), counteract the ET-1/ET_A_ contractile effect by their relaxing effect (functional antagonism). Stimulation of TRPA1-channels by allyl isothiocyanate (AITC) or of TRPV1-channels by capsaicin (CAPS) or rutaecarpine (RUT) leads to release of CGRP. In addition to functional antagonism, stimulation of post junctional CGRP-receptors causes dissociation of ET-1/ET_A_-receptor complexes resulting in termination of thepersistent vasoconstrictor effect of ET-1.

Ligand-binding studies and analyses of structure-affinity and structure-selectivity relationships previously indicated quasi-irreversible and polyvalent binding of ET-1 to ET_A_-receptors[2], [5], [15], [17], [44], [45]. The high affinity of ET-1 for ET_A_-receptors is due to slow dissociation of the agonist-receptor complexes[2]. ET-1 requires the C-terminal Trp^21^, both disulphide bonds and distinct amino acids in the N-terminal loop for high affinity binding to ET_A_-receptors[2], [5], [12], [13], [14], [15], [18]. It has therefore been proposed that several parts of ET-1 interact with distinct sites on the ET_A_-receptor[15], [17]. To the best of our knowledge, the consequences of this polyvalent and irreversible binding of ET-1 to ET_A_-receptors for signaling have not been addressed before. We show that ET_A_-antagonists can prevent binding and contractile effects of ET-1 but that they are less effective in reversing effects induced by ET-1. This discrepancy has also been observed in vivo (e.g. [22]) and was even more marked in an in vitro study using another ET_A_-antagonist [46]. In addition, we report that ET-receptor antagonists reduce not only responses in presence of ET-1 but also responses that had been initiated by ET-1 and that persisted in absence of free agonist. These findings combined with earlier models of ET_A_-receptor function [15], [17] can be integrated into a model regarding ET-1/ET_A_-interactions and ET_A_-mediated signaling as depicted in Fig. 7. A part of ET-1, and the low molecular weight antagonists, binds with high affinity to one binding site on the receptor (site H). Thereafter another part of the ET-1 molecule binds to a second distinct binding-site on the receptor (site L). Binding of ET-1 at site H is dynamic and remains susceptible to competition by the low molecular weight antagonists. It precedes and is required for binding at site L which (i) is insensitive to antagonists, (ii) triggers signalling and (iii) binds the agonist quasi-irreversibly. This model explains the lower potency than affinity and the steepness of the concentration-effect relationships of ET-1 when signaling by ET_A_-receptors is enhanced by cooperativity between the two binding sites of ET-1. In addition, it takes into account the flexibility of ET-1 as indicated by X-ray crystallography and NMR spectroscopy studies [47], [48] and displays similarities to the “address and message domain model” proposed for other GPCR agonists [49], [50]. Because similar findings were obtained with BQ123, SB234551 and bosentan which represent i) hydrophilic and lipophilic antagonists and ii) ET_A_-selective and mixed antagonists, internalization and heterodimerization of receptors do not seem to be involved.

Our model predicts that compounds which accelerate dissociation of ET-1/ET_A_-receptor complexes have a larger and more long-lasting inhibitory effect on responses initiated by ET-1 compared to neutral competitive antagonists. Aspirin-like molecules were reported to display such an allosteric inhibitory effect at millimolar concentrations[45], [51]. We focused on the endothelium and on SMN, two structures that counterbalance ET-1/ET_A_-effects in vivo [4], [7], [25], [29], [30], [31], to identify a similar but more potent mechanism. The endothelium-dependent vasodilator acetylcholine and several directly acting vasodilators fully relaxed ET-1-induced contractions. However, these relaxations were transient and did not inhibit the persistent contractile effect initiated by ET-1. This indicates mere functional antagonism. In contrast, stimuli of SMN not only reversed ET-1-induced contractions but also prevented their recovery. This was observed with capsaicin (pungent vanilloid TRPV1-stimulus of *Capsicum Spec* [40]), rutaecarpine (an alkaloid TRPV1-agonist from the chinese traditional medicinal herb *Evodia Rutaecarpa* [39]) and with allyl isothiocyanate (an organosulfur TRPA1-channel activator of *Allium* and *Brassica* [37]). For rutaecarpine the effects were not modified by removal of the endothelium excluding a role for endothelial TRPV1-channels[52]. The effects of SMN-stimuli were reduced by CGRP-receptor antagonists and mimicked by exogenous CGRP, a neurotransmitter that can be released from peri-arterial SMN [27], [36], [40]. However, they could not be reproduced by agents that stimulate adenylyl cyclase (forskolin and isoproterenol), generate NO (Na-nitroprusside) or open K_ATP_-channels (pinacidil) and thus activate components of the classical signal-transduction mechanism triggered by CGRP-receptors (for review see [53]). Furthermore, the contractile potency and efficacy of ET-1 were hardly modified by pre-exposure to capsaicin or CGRP or after “termination” of the persistent effect of ET-1 by CGRP. This suggests that the cAMP-independent effect of CGRP against ET-1 involves dissociation of ET-1/ET_A_-receptor complexes and not a long-lasting relaxing effect. Clearly, this invites for further investigations into the molecular mechanism(s) induced by CGRP in this setting. These studies should focus on possibilities like i) heterodimers between ET_A_- and CGRP-receptors, ii) rapid phosphorylation followed by desensitization of ET_A_-receptors mediated by e.g. G protein receptor kinases [54], which can be activated by CGRP-receptor stimulation [55] and iii) possible interactions between the different subunits of CGRP receptors, most notably receptor activity modifying protein 1, and ET_A_-receptors. We used imaging to study the effects of CGRP-receptor activation on binding of ET-1 to arterial smooth muscle ET_A_-receptors. In line with earlier findings, fluorescent labeling of ET-1 at Lys^9^ did not modify the pharmacology of the agonist [2], [56], [57]. We observed intense staining of intact vascular smooth muscle which could be prevented by ET-1 and by combined ET_A_- and ET_B_-antagonism ([2] and this study). Thus, we show that ET-1 agonist-receptor binding can be visualised in a vital tissue without the need for supra-physiological receptor densities. Compared to conventional radioligand binding experiments with microsomes or intact arteries (e.g. [58] it has the added value that (i) small tissue samples can be used efficiently without the need for large numbers of arteries and animals, (ii) dissociation of ET-1/ET_A_-complexes can be monitored in real-time and (iii) effects of second messengers and endogenously released mediators (e.g. neurotransmitters) can be registered. In line with the model that we propose, labeling of vascular smooth muscle persisted after removal of free label. In addition, labeling was not reversed by BQ123 in the presence of BQ788. In contrast, capsaicin and CGRP each abolished pre-existing labeling. Thereafter, Rh-ET-1 could again label the smooth muscle with comparable intensity. This strengthens the conclusion that CGRP-receptor stimulation promotes dissociation of the agonist ET-1 from contractile ET_A_-receptors.

In summary (Fig. 7), CGRP released from SMN promotes the dissociation of the ET-1/ET_A_-receptor complexes that are responsible for the long-lasting effects of the peptide. Hence CGRP can be more suited to inhibit vascular effects of ET-1 compared to functional antagonists and competitive antagonists. We could not demonstrate that this mechanism acts as a negative feedback under normal conditions because desensitization of SMN and presence of CGRP-receptor antagonists do not alter the sensitivity to ET-1 ([33] and this study). This is in line with observations that ET-1 does not directly stimulate but modulates effects of TRP channel activators[27], [28]. The negative feedback may become operative during ischemia and inflammation which stimulate SMN activity. Several other aspects remain to be addressed to validate CGRP-receptor agonism and SMN as valid targets for therapy of ET-1-related diseases. These include effects of SMN and CGRP against endogenously produced ET-1 in other vessels and other species. In the mean time it may be worthwhile to consider how widely available natural and orally active stimuli of SMN could be applied in diseases that involve ET-1 but in which clinical efficacy of ET-receptor antagonists has been hard to prove [6], [23].

## Materials and Methods

Experimental protocols were approved by the Ethics Committee on Experimental Animal Welfare of Maastricht University.

### Solutions and Drugs

Bosentan[19], BIBN4096BS[42] and SB-234551[21] were obtained from Actelion Pharmaceuticals (Allschwill, CH), Boehringer Ingelheim Pharma KG (Biberach, D) and GlaxoSmithKline (Stevenage, UK) respectively, and dissolved in DMSO. Allyl isothiocyanate[37], capsaicin[38], [40], forskolin and indomethacin were purchased from Sigma Aldrich (Zwijndrecht, NL) and dissolved in ethanol. Acetylcholine, isoproterenol, L-NAME (Nω (G)-nitro-L- arginine methyl ester), Na-nitroprusside, norepinephrine, and isoproterenol were purchased from Sigma Aldrich (Zwijndrecht, NL) and dissolved in Krebs-Ringer bicarbonate (KRB) solution. Pinacidil was obtained from Sigma Aldrich (Zwijndrecht, NL) and dissolved in DMSO. BQ123[20] and BQ788[34] were obtained from Bachem (Weil am Rhein, D) and dissolved in DMSO. Human CGRP, CGRP _8-37_ [^41^], ET-1 and Ala_1,3,11,15_-ET-1[10] were obtained from Bachem (Weil am Rhein, D) and dissolved in KRB solution. Rutaecarpine[39] was a kind gift from Prof. Yu Huang (Chinese University of Hong Kong, China) and was dissolved in DMSO. The maximal concentrations of the solvents never exceeded 0.1% and did not alter arterial reactivity.

### Tissue Preparation

16 weeks old male WKY rats (Charles River, Maastricht, NL) were euthanized by CO_2_ inhalation. Second-order side branches of the superior mesenteric artery were isolated, and either mounted in a wire-myograph and stretched as previously described [33], [59] or mounted in a pressure-myograph and pressurized at 80 mm Hg[27], [60]. In some arteries, the endothelium was mechanically removed[27], [33], [59].

### Vasomotor responses

At optimal diameter (340±6 µm) the contractile response to 10 µM NE averaged 4.1±0.2 N/m. The relaxing responses to acetylcholine (10 µM) during this precontraction averaged 93.7±0.7% and was absent in denuded arteries.

#### Effects of ET-receptor antagonists

The effect of the ET_A_-antagonists BQ123[20] (1 µM), bosentan[19] (3 µM, in presence of BQ788 (1 µM)) or SB234551[21] (10 nM) was assessed when applied 20 min. before ET-1 induced contractions (0.25–16 nM). In addition, the effect of increasing concentrations (0.1–3 µM) of BQ123 and bosentan was assessed during contractions induced by 8 nM ET-1. Also, the effect of SB234551 (10 nM) during ET-1-induced contraction (8 nM) was determined. Finally, the effect of the antagonists was determined during contractions that remained after removal of ET-1 from its biophase. Before these experiments, peri-arterial SMN were desensitized[33], [38], [40]. In addition, L-NAME (100 µM) and indomethacin (10 µM) were continuously present.

#### Effects of candidate functional antagonists

During ET-1-induced contractions (16 nM), and during contractions that remained after removal of ET-1 (16 nM) from its biophase, arterial relaxing responses to increasing concentrations acetylcholine (0.01–10 µM), capsaicin (0.01–1.0 µM), CGRP (0.1–100 nM), Na-nitroprusside (0.01–10 µM), rutaecarpine (0.1–10 µM), allyl isothiocyanate (0.01–10 µM), forskolin (0.1–3 µM), isoproterenol (0.01–3 µM) or pinacidil (0.01–10 µM) were assessed. These experiments were performed in absence of pharmacological inhibitors and were repeated in presence of L-NAME (100 µM) and indomethacin (10 µM) and in presence of CGRP-receptor antagonists (BIBN4096BS[42] (20 nM) or αCGRP_8-37_ [_41_] (1 µM)). Some of these experiments were repeated in denuded arteries.

### Synthesis of fluorescently labeled ET-1

0.35 mg ET-1 (0.14 µmol) was dissolved in 50 µL dimethylformamide +1 µL N,N-diisopropylethylamine. 50 µL Rhodamine-succinimidyl ester (Rh-SE) stock solution (6.3 µmol/µl 33% acetonitril/67% methanol) was added and left overnight for coupling. After 16 hours HPLC and MALDI-TOF analyses showed that >90% of ET-1 was mono-labeled. Rh-ET-1 was purified using semi-preparative reversed-phase HPLC using a Vydac C-18 column (250×10 mm, 10 µm). A lineair gradient of acetonitrile in water/0.1% TFA (flow rate 5 ml/min; 0.5%B/min) was applied to elute peptides. Rh-ET-1 was lyophilized and stored at −20°C until use.

### Two-photon laser scanning microscopy (TPLSM)

After isolating and pressurizing the arteries, TPLSM was performed as previously described [27], [60]. In short, tissue samples were excited with Tsunami Ti:sapphire laser (Spectra-Physics), which was pumped by a Millennia Vs 5 W pump laser (Spectra-Physics) and mode locked at 840 nm, with a 82.5 MHz repetition rate and 100 fs pulse width. Autofluoresence was visualized at 400 to 450 nm and focal planes were positioned within the tunica media. Arteries were incubated with Rh-ET-1 (16 nM) and labeling of structures in the vessel wall was assessed at 620 to 660 nM. Subsequently, the effect of preincubation with BQ123 (1 µM), BQ123 (1 µM) + BQ788 (1 µM) on labeling was determined. Labeling of arterial smooth muscle in the arterial wall by Rh-ET-1 (16 nM) can be prevented by ET-1 (16 nM)[2]. Finally, the effects of BQ123 (1 µM), removal of free label and antagonist and of administration of either CGRP (100 nM) or of capsaicin (1 µM) on labeling were determined. These experiments were performed in presence of BQ788 (1 µM)[2].

### Data and Statistical Analysis

Contractile responses are expressed as percentage of the maximal contractile response to 10 µM NE in absence of pharmacological inhibitors (NEmax). Relaxing responses are expressed as percentage reduction of the level of pre-contraction. Concentration-response curves (CRC) were fitted to a non-linear sigmoid regression curve (Graphpad Prism 5.0). All data are shown as mean ± SEM. Statistical significance was assessed using either one-way ANOVA (comparison of EC_50_ and E_max_) or two-way ANOVA (comparison of CRCs). Bonferroni's post-hoc test was used to compare multiple groups. A *P* value <0.05 was considered statistically significant.

## Supporting Information

Figure S1 Partial and reversible reversing effect of the ET_A_-receptor antagonist SB234551 on arterial contractile responses to ET-1 and their persistence. Isolated rat mesenteric resistance arteries were studied after treatment with capsaicin (1 µM during 20 min.) in the continuous presence of L-NAME (100 µM) and indomethacin (10 µM). A, responses to 0.25−16 nM ET-1 in the absence (black) and presence of SB234551 (10 nM, red). Note that SB234551 prevented responses to up to 8 nM ET-1. B, vasomotor tone after removal of free agonist and antagonist. C, effects of SB234552 (10 nM) on contractile responses to 8 nM ET-1. D, vasomotor tone after removal of free agonist and antagonist. E, effect of SB234551 (10 nM) on the contractile response initiated by 8 nM ET-1 and persisting in the absence of the peptide. F, vasomotor tone after removal of free antagonist. Data are expressed as % of the maximal response to norepinephrine (NEmax) prior to exposure to any drug, and are shown as mean ± SEM (n = 6). *, the difference from control is statistically significant (P<0.05).
(9.27 MB TIF)

Figure S2 Effects of CGRP and rutaecarpine are endothelium-independent. Isolated, denuded rat arteries were studied in presence of L-NAME (100 µM) and indomethacin (10 µM) as indicated. Arteries were precontracted with 16 nM ET-1. Next, increasing concentrations of vasodilator compounds were administered until a maximal effect was observed. Thereafter vasoactive stimuli were removed from the organ chamber while the recording of active wall tension continued for >10 min. A, maximal acute relaxing effects of CGRP and rutaecarpine. B, long-term effects of CGRP and rutaecarpine. a, time control; b, CGRP; c, rutaecarpine.
(8.27 MB TIF)
