# Supplementary figures and images for: Stimuli of Sensory-Motor Nerves Terminate Arterial Contractile Effects of Endothelin-1 by CGRP and Dissociation of ET-1/ETA-Receptor Complexes
Source: PLoS One. 2010 Jun 1;5(6):e10917. doi: 10.1371/journal.pone.0010917 (PMC2879375; doi:10.1371/journal.pone.0010917)

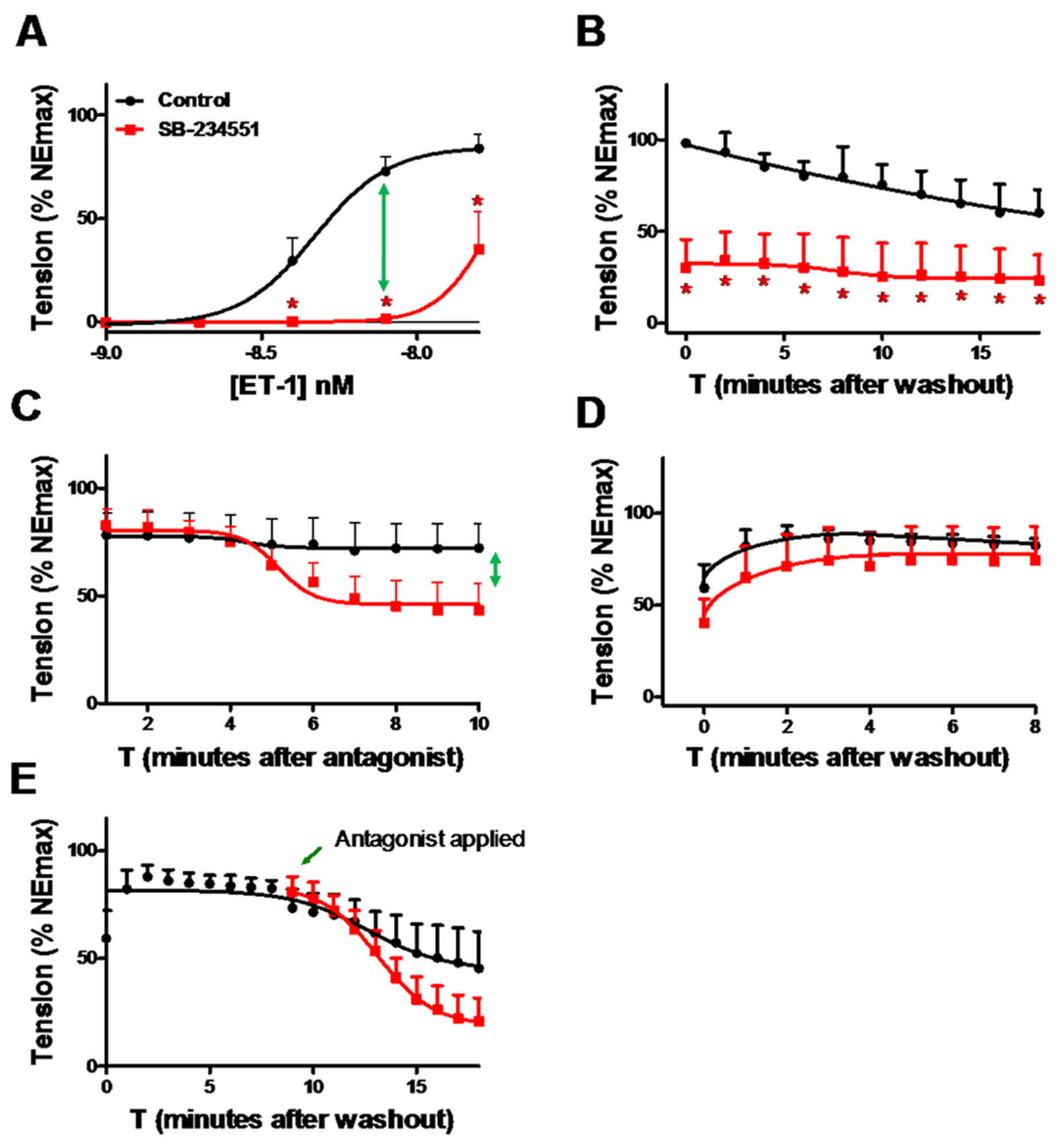

Supplement: Figure S1 — Partial and reversible reversing effect of the ETA-receptor antagonist SB234551 on arterial contractile responses to ET-1 and their persistence. Isolated rat mesenteric resistance arteries were studied after treatment with capsaicin (1 µM during 20 min.) in the continuous presence of L-NAME (100 µM) and indomethacin (10 µM). A, responses to 0.25−16 nM ET-1 in the absence (black) and presence of SB234551 (10 nM, red). Note that SB234551 prevented responses to up to 8 nM ET-1. B, vasomotor tone after removal of free agonist and antagonist. C, effects of SB234552 (10 nM) on contractile responses to 8 nM ET-1. D, vasomotor tone after removal of free agonist and antagonist. E, effect of SB234551 (10 nM) on the contractile response initiated by 8 nM ET-1 and persisting in the absence of the peptide. F, vasomotor tone after removal of free antagonist. Data are expressed as % of the maximal response to norepinephrine (NEmax) prior to exposure to any drug, and are shown as mean ± SEM (n = 6). *, the difference from control is statistically significant (P<0.05). (9.27 MB TIF) [file pone.0010917.s001.tif]

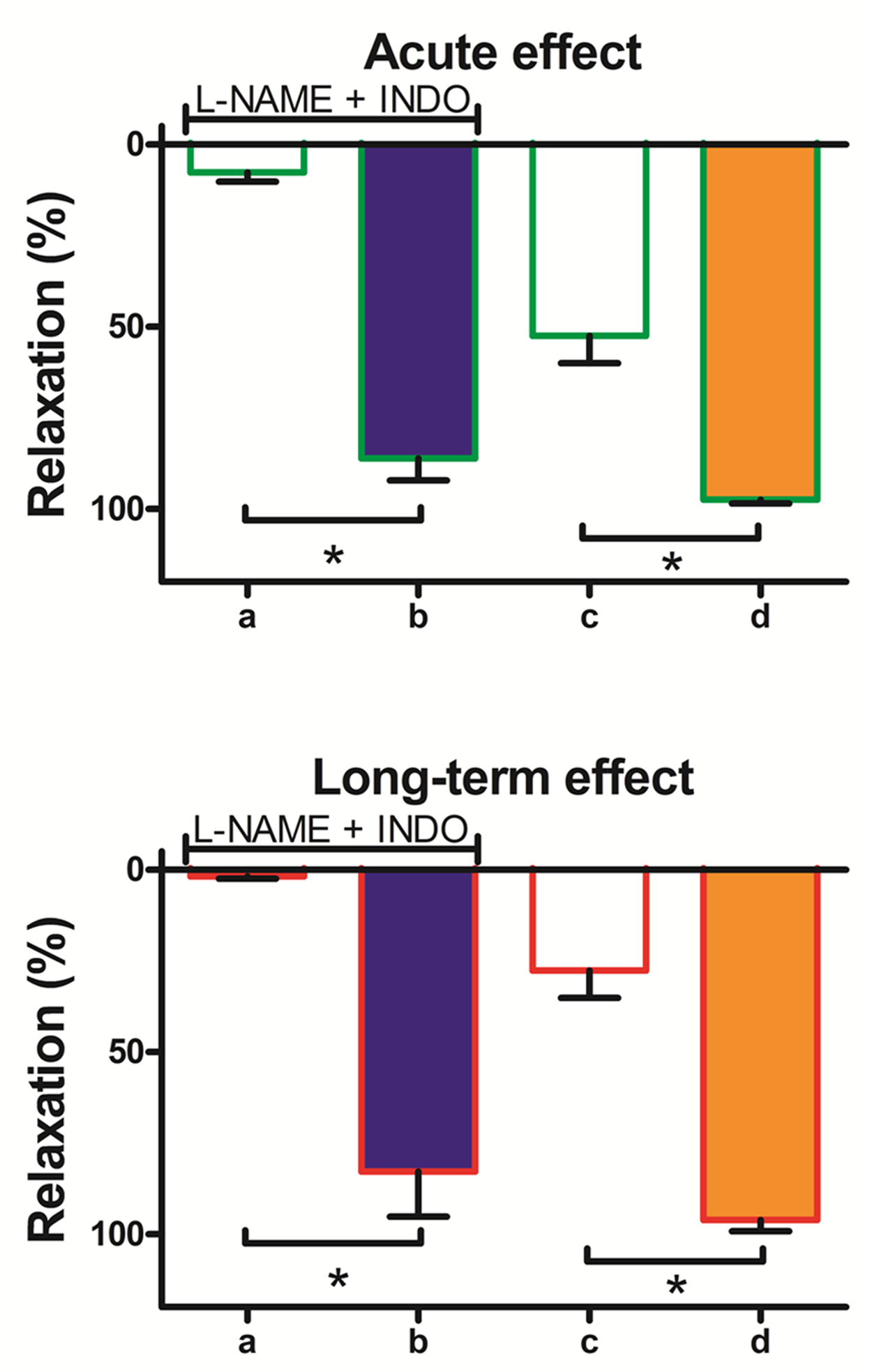

Supplement: Figure S2 — Effects of CGRP and rutaecarpine are endothelium-independent. Isolated, denuded rat arteries were studied in presence of L-NAME (100 µM) and indomethacin (10 µM) as indicated. Arteries were precontracted with 16 nM ET-1. Next, increasing concentrations of vasodilator compounds were administered until a maximal effect was observed. Thereafter vasoactive stimuli were removed from the organ chamber while the recording of active wall tension continued for >10 min. A, maximal acute relaxing effects of CGRP and rutaecarpine. B, long-term effects of CGRP and rutaecarpine. a, time control; b, CGRP; c, rutaecarpine. (8.27 MB TIF) [file pone.0010917.s002.tif]
